# Three-Year Functional Vision, Longitudinal Refractive Outcomes, and Patient-Reported Outcomes After Bilateral Implantation of the Extend Bessel-Optics Intraocular Lens: An Independent Real-World Ambispective Study

**DOI:** 10.3390/medicina62101883

**Published:** 2026-09-29

**Authors:** Germán Roberto Bianchi, Sofía Lorenzetti

**Affiliations:** Clínica de Ojos Dr. Nano Buenos Aires Blas Parera 4201, Olivos B1636CSS, Provincia de Buenos Aires, Argentina; solorenzetti@gmail.com

**Keywords:** Bessel optics, Partial-DOFi Extended, intraocular lens, cataract surgery, longitudinal refractive outcomes, defocus curve, patient-reported outcomes, spectacle independence

## Abstract

*Background and Objectives*: Independent evidence on the longer-term performance of the Extend Bessel-optics intraocular lens remains limited. This study aimed to evaluate refractive and visual changes beyond the first postoperative year and to characterize binocular functional vision, defocus performance, and patient-reported outcomes 36–48 months after bilateral implantation. *Materials and Methods*: This ambispective, single-center case series included patients who had undergone bilateral implantation of the non-toric Extend IOL with emmetropic targets and had complete one-year postoperative data. One-year refraction and visual acuity data were retrieved retrospectively; eligible patients who had reached at least 36 months after surgery underwent prospective evaluation including refraction, visual acuity, defocus curves, pupillometry, VF-14, spectacle use, dysphotopsia, and satisfaction. *Results*: Of 37 patients in the source cohort, two lacked the required one-year postoperative visit. All 35 eligible patients (70 eyes) completed the prospective evaluation at 36–48 months. No statistically significant change in subjective spherical equivalent was detected (mean paired change, +0.029 D; 95% CI, −0.005 to +0.062 D; *p* = 0.114). Small statistically significant changes occurred in objective spherical equivalent (−0.052 D) and UDVA, UIVA, and UNVA (+0.009, +0.027, and +0.025 logMAR, respectively). Mean binocular defocus acuity remained ≤0.20 logMAR from +1.00 to −2.00 D, and the pooled monocular profile was compatible with a Partial-DOFi Extended functional classification. Mean VF-14 score was 98.9 ± 2.5; 94.3–97.1% of patients reported never using spectacles across viewing distances, and dysphotopsia was infrequent and predominantly mild. *Conclusions*: Subjective spherical equivalent showed no statistically significant change between one year and 36–48 months, whereas small statistically significant changes occurred in objective refraction and uncorrected visual acuity. Cross-sectional functional and patient-reported outcomes at 3–4 years were favorable in this selected cohort, which was characterized by relatively high implanted IOL powers.

## 1. Introduction

Cataract surgery has evolved from a procedure aimed primarily at restoring lens transparency into a refractive intervention intended to optimize postoperative visual function and quality of life [1,2,3,4,5,6]. Contemporary surgical success is no longer defined solely by excellent distance visual acuity, but by the patient’s ability to perform everyday activities comfortably across multiple viewing distances while minimizing spectacle dependence [5,7]. As life expectancy increases and older adults remain professionally, socially, and recreationally active, cataract surgery has become an integral component of lifestyle-oriented visual rehabilitation, in which visual needs, daily activities, and patient expectations play a central role in intraocular lens (IOL) selection [7,8,9,10].

Contemporary presbyopia-correcting and range-extending intraocular lenses have historically been described using optical or commercial labels such as multifocal, extended depth-of-focus (EDOF), enhanced-monofocal, and monofocal-plus platforms [1,2,3,4,11,12]. More recently, functional classification has shifted toward performance measured with standardized monocular distance-corrected defocus curves. The 2026 Global Functional Vision Working Group consensus recommends the term depth of field (DOFi) and classifies lenses as Partial-DOFi (Narrowed, Enhanced, or Extended) or Full-DOFi (Steep, Smooth, or Continuous) according to prespecified defocus-curve thresholds [7,13]. This functional framework is intentionally distinct from manufacturer terminology and from the optical mechanism used to generate the observed range of vision.

Modern IOL selection also extends beyond optical performance alone. Current evidence-based frameworks emphasize matching the probability of achieving specific functional visual goals to each patient’s visual priorities, occupational and recreational demands, tolerance to dysphotopsia, ocular characteristics, and economic considerations [7,8,9,10,13]. Consequently, the most appropriate IOL is not necessarily the most technologically complex platform, but the one that provides an acceptable balance among distance image quality, functional range, visual symptoms, and patient expectations.

Within this context, the Extend intraocular lens (Hanita Lenses, Kibbutz Hanita, Israel) is a non-diffractive Bessel-optics focal-extension platform intended to enhance intermediate vision while preserving a distance-dominant optical profile [14,15]. Its refractive design incorporates a central axicon-like component intended to generate an elongated Bessel-like focal region rather than multiple discrete diffractive foci. Because commercial or design descriptors and functional classifications answer different questions, this manuscript refers to the device neutrally as the Extend Bessel-optics IOL and separately reports its observed functional profile according to the current DOFi framework.

A targeted PubMed/MEDLINE search, last updated on 1 September 2026, using the terms “Extend intraocular lens” and “Bessel intraocular lens” identified peer-reviewed optical-bench evidence and shorter-term clinical reports or conference presentations, but no prior independent peer-reviewed clinical report with three-year follow-up of this specific platform. The novelty claim in this manuscript is therefore limited to independent peer-reviewed long-term clinical follow-up rather than to the optical concept itself.

Visual performance after implantation of focal-extension intraocular lenses may also be influenced by patient-specific ocular characteristics. Among these, pupil diameter has received increasing attention because it may affect effective depth of focus, retinal image quality, and overall visual performance depending on the optical architecture of the implanted lens [12]. These observations further support the concept that successful refractive cataract surgery depends not only on IOL design but also on appropriate patient selection.

The present study therefore evaluated longitudinal changes in refraction and uncorrected visual acuity between one year and long-term follow-up after bilateral Extend implantation, together with cross-sectional binocular visual function, defocus performance, pupil characteristics, and patient-reported outcomes at the long-term evaluation. We hypothesized that no large longitudinal refractive change would be detected and that long-term functional and patient-reported outcomes would be favorable in this clinically selected cohort.

## 2. Materials and Methods

### 2.1. Study Design and Setting

This ambispective, non-randomized, non-comparative, single-center case series was conducted at Clínica de Ojos Dr. Nano, Buenos Aires, Argentina. Institutional records were reviewed to identify patients who had undergone bilateral cataract surgery with implantation of the Extend Bessel-optics IOL (Hanita Lenses, Kibbutz Hanita, Israel) between March 2022 and March 2023, met the eligibility criteria, and had complete one-year postoperative data. Patients who had reached at least 36 months after surgery were contacted and invited to return for a prospective long-term evaluation. One-year refraction and visual-acuity data were obtained retrospectively, whereas the long-term visit prospectively assessed refraction, visual acuity, defocus performance, pupillometry, and patient-reported outcomes. Defocus curves, pupillometry, and patient-reported outcomes were obtained only at the long-term visit. The study was conducted in accordance with the Declaration of Helsinki and was approved by the Institutional Review Board of Clínica de Ojos Dr. Nano (approval GBCN01/25). All participants provided written informed consent before the prospective long-term evaluation.

### 2.2. Study Population

Extend implantation had been performed as part of routine clinical practice before the study was conceived. Patients were eligible if they had undergone bilateral implantation of the same non-toric Extend model, both eyes had been targeted for emmetropia, complete one-year postoperative data were available, and at least 36 months had elapsed since surgery. Exclusion criteria comprised ocular comorbidities potentially affecting postoperative visual performance (including advanced glaucoma, retinal disorders, corneal pathology, or optic neuropathy), previous ocular surgery other than uncomplicated cataract extraction, intraoperative complications, and postoperative complications other than posterior capsule opacification that could materially affect visual outcomes.

### 2.3. Preoperative Evaluation, Refractive Target, and Surgery

Before cataract surgery, all patients had undergone the center’s routine comprehensive ophthalmic evaluation and optical biometry for IOL power calculation. Both eyes were targeted for emmetropia; no monovision strategy was planned, and ocular dominance was not used to select different refractive targets. All surgeries were performed by a single surgeon (G.R.B.) using standard phacoemulsification with in-the-bag implantation of the Extend IOL.

### 2.4. Intraocular Lens

The Extend IOL (Hanita Lenses, Kibbutz Hanita, Israel) is a single-piece hydrophobic acrylic intraocular lens with a 6.0 mm optic and an overall diameter of 13.0 mm. Its purely refractive, non-diffractive optic incorporates a modified higher-order aspheric surface with a central axicon-like phase profile. Within the finite aperture of the IOL, this design is intended to generate a finite-length Bessel-like focal region and modest focal elongation without dividing incoming light among discrete diffractive foci [14,15]. According to the manufacturer specifications, the central optical power increases by approximately +1.20 D and progressively decreases toward the peripheral nominal power, aiming to improve intermediate vision while preserving distance performance [14]. The lens incorporates ultraviolet- and violet-light filtering and is available in spherical and toric configurations.

### 2.5. Outcome Measures

The primary longitudinal outcome was change in subjective spherical equivalent between the one-year and long-term postoperative evaluations. Secondary longitudinal outcomes were changes in objective spherical equivalent, uncorrected distance visual acuity (UDVA), uncorrected intermediate visual acuity (UIVA, 66 cm), and uncorrected near visual acuity (UNVA, 40 cm). The Outcomes assessed only at the long-term visit included monocular and binocular distance, intermediate, and near visual acuity, monocular and binocular defocus curves, posterior capsule status, VF-14, spectacle independence, dysphotopsia, satisfaction, and pupillometry. Exploratory outcomes included associations between photopic and mesopic pupil diameter and long-term refractive, visual, defocus, and patient-reported outcomes.

### 2.6. Clinical Assessment

At the prospective long-term visit, all patients underwent manifest refraction, uncorrected and corrected visual acuity assessment, slit-lamp biomicroscopy, assessment of IOL centration and posterior capsule transparency, and dilated fundus examination. Visual acuity was measured using standardized Early Treatment Diabetic Retinopathy Study (ETDRS) charts and recorded in logMAR units. Subjective spherical equivalent was calculated as sphere plus half the cylindrical power. Objective refraction was measured using an autorefractometer (Topcon KR-800, Topcon Medical Japan Co., Ltd., Tokyo, Japan), and objective spherical equivalent was calculated similarly. Intermediate and near visual acuity were tested at 66 cm and 40 cm, respectively. IOL centration and posterior capsule transparency were assessed clinically at the slit lamp. Photopic and mesopic pupil diameters were measured using the Pentacam HR imaging system (OCULUS Optikgeräte GmbH, Wetzlar, Germany) under standardized illumination conditions. The one-year examinations were performed using the same ETDRS charts, testing distances, illumination conditions, manifest-refraction protocol, Topcon KR-800 autorefractometer, and clinical examination protocol.

### 2.7. Defocus Curve

Monocular and binocular defocus curves were obtained only at the prospective long-term visit with best distance correction in place. Visual acuity was measured under standardized photopic conditions in 0.50-D steps from +3.00 to −4.00 D. Mean visual acuity at each defocus level was used to describe the functional through-focus profile. For functional classification, the pooled mean monocular curve was normalized to the plano value, and the negative-defocus crossings at 0.20 and 0.30 logMAR were estimated by linear interpolation according to the 2026 Global Functional Vision Working Group framework [13].

### 2.8. Patient-Reported Outcomes

Patient-reported outcomes were assessed only at the prospective long-term visit. Visual function was assessed using the VF-14 questionnaire originally developed by Steinberg et al. [16]. The questionnaire was administered in Spanish using the version evaluated in the international validation study that included the Barcelona cohort [17], and scored from 0 to 100, with higher scores indicating better visual function. Patients also completed a study-specific structured questionnaire assessing spectacle independence, visual phenomena, overall satisfaction, and willingness to undergo the same procedure again. Visual phenomena were graded as none, mild, moderate, or severe. Spectacle use was recorded separately for distance, intermediate, and near vision as never, sometimes, or nearly always. Overall satisfaction was recorded as very satisfied, satisfied, less satisfied, or unsatisfied, and willingness to undergo the same procedure again as yes or no. The questionnaire domains and response categories are provided in Supplementary Table S1.

### 2.9. Statistical Analysis

Continuous variables are presented as mean ± standard deviation (SD), median, and range, as appropriate. Categorical variables are presented as frequencies and percentages. Descriptive ocular outcomes were summarized at the eye level. For longitudinal comparisons, the patient was the primary unit of analysis: values from both eyes were averaged for each patient at each time point [18,19]. Normality of paired differences was assessed using the Shapiro–Wilk test. Because paired differences were not normally distributed, one-year and long-term outcomes were compared using the Wilcoxon signed-rank test. Mean paired changes are also reported with 95% confidence intervals.

As a sensitivity analysis, longitudinal outcomes were additionally analyzed at the eye level using generalized estimating equations (GEE) with patient as the clustering variable. A Holm adjustment was applied as a multiplicity sensitivity analysis to the four secondary longitudinal outcomes. Binocular visual acuity, binocular defocus curves, VF-14, spectacle independence, dysphotopsia, satisfaction, and pupillometry were analyzed at the patient level. Exploratory associations between pupil diameter and postoperative outcomes were assessed using Spearman rank correlation coefficients without adjustment for multiple comparisons and were considered hypothesis-generating.

A two-sided *p* value < 0.05 was considered statistically significant. No a priori sample-size calculation was performed because the study evaluated a fixed pre-existing clinical cohort; the final sample size was therefore determined by the number of patients in this cohort who met the prespecified eligibility criteria, rather than by prospective recruitment to a target sample size. Post hoc observed-power calculations were not used to reinterpret completed-study findings [20]. No imputation was required because the reported analyses had complete data. Primary analyses were performed using XLMiner Analysis ToolPak for Microsoft Excel (Frontline Systems Inc., Incline Village, NV, USA); GEE sensitivity analyses were performed in Python 3.13 using statsmodels 0.14.6.

## 3. Results

### 3.1. Study Population

The source clinical cohort comprised 37 patients with bilateral Extend implantation during the study period. Two patients had not completed the routine one-year postoperative visit and therefore lacked the longitudinal data required for study eligibility; these patients were excluded before prospective recall. The remaining 35 patients (70 eyes) had complete one-year data, had reached at least 36 months after surgery, and were eligible for the prospective long-term evaluation. All 35 eligible patients were invited, agreed to participate, and completed the study visit, corresponding to a 100% participation rate among eligible patients. Postoperative follow-up ranged from 36 to 48 months; four patients were evaluated at 48 months, and the remaining patients were evaluated between these time points. Mean patient age was 68.9 ± 8.4 years (range, 54–85 years); 18 patients were female (51.4%), and 17 were male (48.6%). Mean implanted IOL power was 26.19 ± 1.86 D (range, 21.50–30.20 D), with a median of 26.16 D (interquartile range, 25.15–27.61 D). Overall, 54 of 70 eyes (77.1%) received an IOL power of ≥25.0 D, 23 eyes (32.9%) received ≥27.0 D, and 14 eyes (20.0%) received ≥28.0 D, further demonstrating that relatively high IOL powers were common in this cohort. No intraoperative complications were identified in the reviewed surgical records.

### 3.2. Clinical Assessment

At the prospective long-term evaluation (36–48 months), slit-lamp biomicroscopy showed clinically centered IOLs in all eyes (Figure 1). No eye had posterior capsule opacification considered clinically significant or requiring Nd:YAG laser capsulotomy. Because no formal quantitative PCO grading system was prespecified, subtle subclinical capsular haze cannot be excluded. Dilated fundus examination revealed no retinal or optic-nerve abnormality considered sufficient to explain reduced visual performance. Mean subjective spherical equivalent was −0.17 ± 0.27 D, with 45 eyes (64.3%) within ±0.25 D, 66 eyes (94.3%) within ±0.50 D, and all eyes within ±1.00 D of emmetropia. Mean objective spherical equivalent was −0.31 ± 0.30 D, with 37 eyes (52.9%) within ±0.25 D, 54 eyes (77.1%) within ±0.50 D, and all eyes within ±1.00 D.

Mean monocular uncorrected and corrected distance visual acuity were 0.04 ± 0.05 logMAR and −0.03 ± 0.04 logMAR, respectively (Figure 2). All eyes achieved 0.10 logMAR or better for both UDVA and CDVA. Mean binocular uncorrected visual acuity was −0.03 ± 0.05 logMAR for distance, 0.12 ± 0.09 logMAR at 66 cm, and 0.26 ± 0.12 logMAR at 40 cm. Figure 2 illustrates postoperative refractive accuracy relative to emmetropia. Complete long-term refractive, visual, binocular, and pupillometry outcomes are summarized in Table 1.

Mean photopic pupil diameter was 2.28 ± 0.13 mm (range, 2.02–2.50 mm), whereas mean mesopic pupil diameter was 2.80 ± 0.22 mm (range, 2.41–3.24 mm). Exploratory analyses showed no statistically significant associations between photopic or mesopic pupil diameter and postoperative refractive outcomes, visual acuity, binocular defocus performance, or patient-reported outcomes.

### 3.3. Longitudinal Refractive Outcomes

Longitudinal refractive and visual outcomes are summarized in Table 2 using patient-level bilateral averages, consistent with the primary inferential analysis. Mean subjective spherical equivalent changed from −0.204 ± 0.208 D at one year to −0.175 ± 0.204 D at long-term follow-up; no statistically significant change was detected (mean paired change, +0.029 ± 0.097 D; 95% CI, −0.005 to +0.062 D; *p* = 0.114). Objective spherical equivalent showed a statistically significant myopic change of −0.052 ± 0.094 D (95% CI, −0.084 to −0.019 D; *p* = 0.003). UDVA, UIVA at 66 cm, and UNVA at 40 cm showed small statistically significant increases in logMAR of +0.009, +0.027, and +0.025, respectively (all unadjusted *p* ≤ 0.002). In a Holm multiplicity sensitivity analysis, adjusted *p* values were 0.0046 for objective spherical equivalent, 0.0046 for UDVA, 0.00016 for UIVA, and 0.00021 for UNVA. The GEE sensitivity analysis, which retained both eyes and accounted for within-patient correlation, produced concordant estimates: +0.029 D for subjective spherical equivalent (*p* = 0.078), −0.052 D for objective spherical equivalent (*p* < 0.001), and +0.009, +0.027, and +0.025 logMAR for UDVA, UIVA, and UNVA, respectively (all *p* ≤ 0.002). Thus, statistically detectable worsening occurred in objective refraction and uncorrected visual-acuity measures, while the magnitude of these changes was small; their clinical relevance cannot be established from statistical significance alone.

### 3.4. Binocular Defocus Curve

The source defocus data were rechecked against Figure 3. The figure was consistent with the final analytic workbook, whereas the previous numerical text overstated the negative-defocus range. Corrected mean binocular visual acuity was −0.031 logMAR at plano and remained 0.20 logMAR or better from +1.00 D (0.187 logMAR) through −2.00 D (0.166 logMAR). Mean binocular acuity then worsened to 0.261, 0.319, 0.354, and 0.500 logMAR at −2.50, −3.00, −3.50, and −4.00 D, respectively. The corrected curve therefore supports a continuous distance-to-intermediate range with additional but progressively reduced near performance rather than the broader range stated previously. After normalization of the pooled mean monocular curve to the plano value and linear interpolation, estimated negative DOFi was approximately 2.06 D at the 0.20-logMAR cutoff and 2.57 D at the 0.30-logMAR cutoff. These values fall within the current Partial-DOFi Extended category (0.20-logMAR DOFi ≥ 1.58 and <2.3 D; 0.30-logMAR DOFi ≥ 1.98 and <2.75 D) [13]. Supplementary Table S2 reports the exact mean OD, OS, and OU values at each defocus level. The retained analytic workbook contains aggregate mean defocus curves rather than individual-level defocus measurements; therefore, SD/error bars cannot be reconstructed from this file alone.

### 3.5. Patient-Reported Outcomes

At the prospective long-term visit (36–48 months), patient-reported outcomes were assessed using the VF-14 and the study-specific structured questionnaire. The mean VF-14 score was 98.9 ± 2.5, with a median of 100 (range, 91.1–100); 28 patients (80.0%) achieved the maximum score of 100 (Table 3). Spectacle use was low at all viewing distances: 33/35 patients (94.3%) selected ‘never’ for distance spectacles, 34/35 (97.1%) for intermediate spectacles, and 33/35 (94.3%) for near spectacles (Table 3). These are cross-sectional long-term findings and were not measured at one year.

At long-term follow-up, visual symptoms were infrequent and predominantly mild. Halos were reported by five patients (14.3%; four mild and one moderate), mild glare by one patient (2.9%), and mild blurred distance vision, blurred near vision, and night-vision difficulty by three patients each (8.6%) (Table 4). No patient reported monocular or binocular diplopia, depth-perception difficulty, or color-vision difficulty. Thirty-four patients (97.1%) were very satisfied, and one (2.9%) was satisfied; all 35 patients stated that they would choose the same procedure again. These satisfaction and symptom outcomes were assessed only at the long-term visit.

## 4. Discussion

The present study evaluated longitudinal refractive and visual change from one year to long-term follow-up and cross-sectional functional and patient-reported outcomes at long-term follow-up after bilateral Extend implantation. No statistically significant change in subjective spherical equivalent was detected; the mean paired change was +0.029 D, and its 95% confidence interval crossed zero. In contrast, objective spherical equivalent showed a small statistically significant myopic change, and UDVA, UIVA, and UNVA showed small statistically significant increases in logMAR. These findings should be described as statistically detectable changes rather than as formal proof of equivalence or stability. Separately, the long-term cross-sectional evaluation showed favorable binocular acuity, a smooth through-focus profile, high VF-14 scores, low spectacle use, and predominantly mild visual symptoms. The longitudinal and cross-sectional findings therefore provide complementary, but not interchangeable, information about long-term performance.

The optical design may help contextualize, but cannot by itself explain, the observed clinical profile. Extend uses a continuous refractive surface with a central axicon-like phase profile intended to create an elongated Bessel-like focal region without diffractive light splitting [14,15,21]. The present clinical study did not measure wavefront propagation, modulation transfer function, or retinal image quality and therefore does not directly demonstrate that the Bessel-like mechanism caused the clinical outcomes. The corrected smooth defocus profile is compatible with focal elongation, but should be regarded as clinical behavior rather than mechanistic proof.

The findings should also be interpreted within the contemporary functional classification framework. The 2026 Global Functional Vision Working Group consensus replaced the earlier RoF terminology with DOFi and classifies IOL performance using the normalized monocular distance-corrected defocus curve [13]. Based on the corrected pooled mean monocular curve in this cohort, estimated negative DOFi was approximately 2.06 D at 0.20 logMAR and 2.57 D at 0.30 logMAR, values compatible with a Partial-DOFi Extended profile rather than the Partial-DOFi Enhanced category. This functional descriptor is separate from the manufacturer’s commercial or design terminology and avoids assuming that a specific optical mechanism necessarily determines a fixed clinical category.

Independent optical-bench evidence provides complementary context. Kremser et al. compared Extend with the Tecnis Eyhance enhanced-monofocal IOL and reported broadly similar through-focus optical performance under their bench conditions [21]. These data help situate Extend among distance-dominant focal-extension strategies, but optical-bench similarity does not establish clinical equivalence, and the present study had no head-to-head comparator.

The primary longitudinal outcome was subjective spherical-equivalent change. No statistically significant change was detected between one year and long-term follow-up, and the 95% confidence interval for the mean paired change was −0.005 to +0.062 D; nevertheless, equivalence was not formally tested. The statistically significant objective myopic shift (−0.052 D) occurred without a corresponding manifest-refraction change. In pseudophakic eyes, this subjective-objective dissociation may reflect autorefractor-related bias, tear-film or alignment effects, or small differences in pupil state rather than a true optical change in the implanted IOL. Importantly, the one-year and long-term visits used the same chart type, test distances, illumination protocol, manifest-refraction procedure, autorefractometer model, and clinical examination protocol, making a systematic protocol change less likely as an explanation. The small statistically significant worsening in UDVA, UIVA, and UNVA (0.009–0.027 logMAR) is reported transparently; its clinical relevance is uncertain because statistical significance alone does not define a clinically meaningful change.

Rechecking the original defocus spreadsheet resolved an important discrepancy between the prior numerical text and Figure 3. Mean binocular acuity remained 0.20 logMAR or better from +1.00 through −2.00 D, not through −3.50 D as previously stated. Beyond −2.00 D, mean acuity worsened progressively. Thus, the corrected curve supports a distance-to-intermediate functional range with additional near extension, but not the substantially broader near range claimed in the original text. This correction also brings the clinical description into closer alignment with the Partial-DOFi Extended range estimated from the monocular curves under the current functional classification [13].

Patient-reported outcomes at the long-term visit add an important patient-centered dimension to the objective measurements. VF-14 scores approached the maximum possible score in most patients, spectacle use was low across viewing distances, and visual symptoms were generally absent or mild. However, these measures were not collected at one year; they therefore describe favorable cross-sectional status at long-term follow-up and should not be interpreted as evidence that satisfaction, spectacle independence, or dysphotopsia remained stable throughout follow-up.

During routine postoperative consultations, some patients spontaneously used descriptors such as ‘natural’ and ‘clear’ for their vision. These comments were not systematically elicited, recorded with a validated quality-of-vision instrument, or prespecified as outcomes and therefore remain anecdotal clinical observations. They are not used as evidence of longitudinal change or as a formal outcome of the study.

Pupil diameter may influence focal-extension optics because aperture size affects depth of field and retinal image quality. No statistically significant associations between pupil diameter and the measured long-term outcomes were detected in this cohort. Given the small sample, narrow pupil-size distribution, and multiple exploratory correlations, these null findings are imprecise and should not be interpreted as evidence that pupil diameter has no effect. Larger cohorts with broader pupil distributions are required.

Direct comparison with other focal-extension platforms is limited by the absence of a comparator arm and by substantial heterogeneity in patient selection, testing distance, defocus-curve methodology, pupil conditions, and patient-reported instruments across published studies. The present findings show qualitative parallels with reports of Symfony, Vivity, PureSee, Isopure, Mini WELL, Lucidis, and other contemporary technologies [10,22,23,24,25,26,27,28,29,30], but these cross-study similarities should not be interpreted as formal comparative evidence or as proof of equivalent performance.

The broader literature, including a recent Cochrane review, suggests that EDOF and other focal-extension strategies can provide strong distance and intermediate performance with different trade-offs in near vision and photic phenomena [31]. Because Extend was not compared directly with a trifocal, conventional monofocal, or another focal-extension lens in this study, the present results cannot determine relative superiority or infer a class-wide benefit.

An important characteristic of the present cohort was the relatively high distribution of implanted IOL powers. Mean IOL power was 26.19 ± 1.86 D, with a median of 26.16 D (interquartile range, 25.15–27.61 D; range, 21.50–30.20 D); 77.1% of eyes received an IOL power of at least 25.0 D and 32.9% received at least 27.0 D. The present findings therefore provide their most direct clinical evidence for eyes requiring IOL powers within this relatively high range. Such powers are commonly encountered in eyes with shorter axial dimensions and/or hyperopic biometric profiles. However, because axial length, keratometry, and preoperative refraction were not retained in the analytic dataset, the cohort cannot be formally classified as a short-eye or hyperopic population. Accordingly, extrapolation specifically to those biometric subgroups should remain cautious.

The present study has several limitations. It was a single-center, non-comparative case series with a fixed cohort of 35 eligible participants and no a priori sample-size calculation, limiting precision and power, particularly for negative exploratory findings. The source cohort comprised 37 bilaterally implanted patients; two were excluded before recall because they had not completed the required one-year postoperative visit, whereas all 35 eligible patients completed the prospective long-term evaluation. Although this complete participation among eligible patients reduces concern about responder bias at the recall stage, requiring an available one-year examination may still introduce selection related to earlier follow-up. Preoperative axial length, keratometry, anterior chamber depth, WTW, calculation-formula details, and baseline refraction/visual acuity were not prespecified long-term study variables and were not included in the analytic dataset. The absence of complete preoperative biometric characterization further limits subgroup-specific generalizability. The ambispective design may also be susceptible to documentation and test–retest variability, although the same clinical measurement protocol was used at one year and at the long-term evaluation. Contrast sensitivity, objective retinal-image quality, and wavefront aberrometry were not evaluated. Posterior capsule status was assessed clinically rather than with a formal quantitative grading system; early or subclinical capsular haze below the threshold for Nd:YAG treatment therefore cannot be excluded as a contributor to small visual-acuity changes. Individual-level defocus data were not retained in the final analytic workbook, preventing retrospective calculation of SD/error bars for Figure 3 from the deposited dataset. Finally, the study-specific questionnaire was not a validated composite quality-of-vision scale.

From a clinical standpoint, the longitudinal data show no statistically significant change in the subjective spherical equivalent but small statistically significant changes in objective refraction and uncorrected visual acuity. The separate long-term assessment shows favorable binocular functional and patient-reported outcomes. Taken together, these results provide a multidimensional description of this selected cohort at long-term follow-up, while avoiding the inference that outcomes measured only at long-term follow-up were necessarily maintained unchanged over the entire postoperative interval.

## 5. Conclusions

In conclusion, no statistically significant change in subjective spherical equivalent was detected between one year and the 36- to 48-month follow-up after bilateral implantation of the Extend Bessel-optics IOL, whereas small statistically significant changes were observed in objective refraction and uncorrected distance, intermediate, and near visual acuity. At the 3- to 4-year cross-sectional evaluation, binocular functional vision, VF-14, spectacle use, satisfaction, and dysphotopsia outcomes were favorable, and the pooled monocular defocus profile was compatible with a Partial-DOFi Extended functional classification. These findings provide independent clinical evidence on the 3- to 4-year performance of the Extend platform in a selected, non-comparative cohort, characterized by relatively high implanted IOL powers, but do not establish equivalence or comparative superiority. The results may therefore be particularly informative for eyes requiring similar higher-power IOLs; however, because axial length and preoperative refraction were unavailable, direct extrapolation specifically to short or hyperopic eyes should remain cautious. Future controlled studies across broader biometric ranges are warranted.

## Figures and Tables

**Figure 1:**
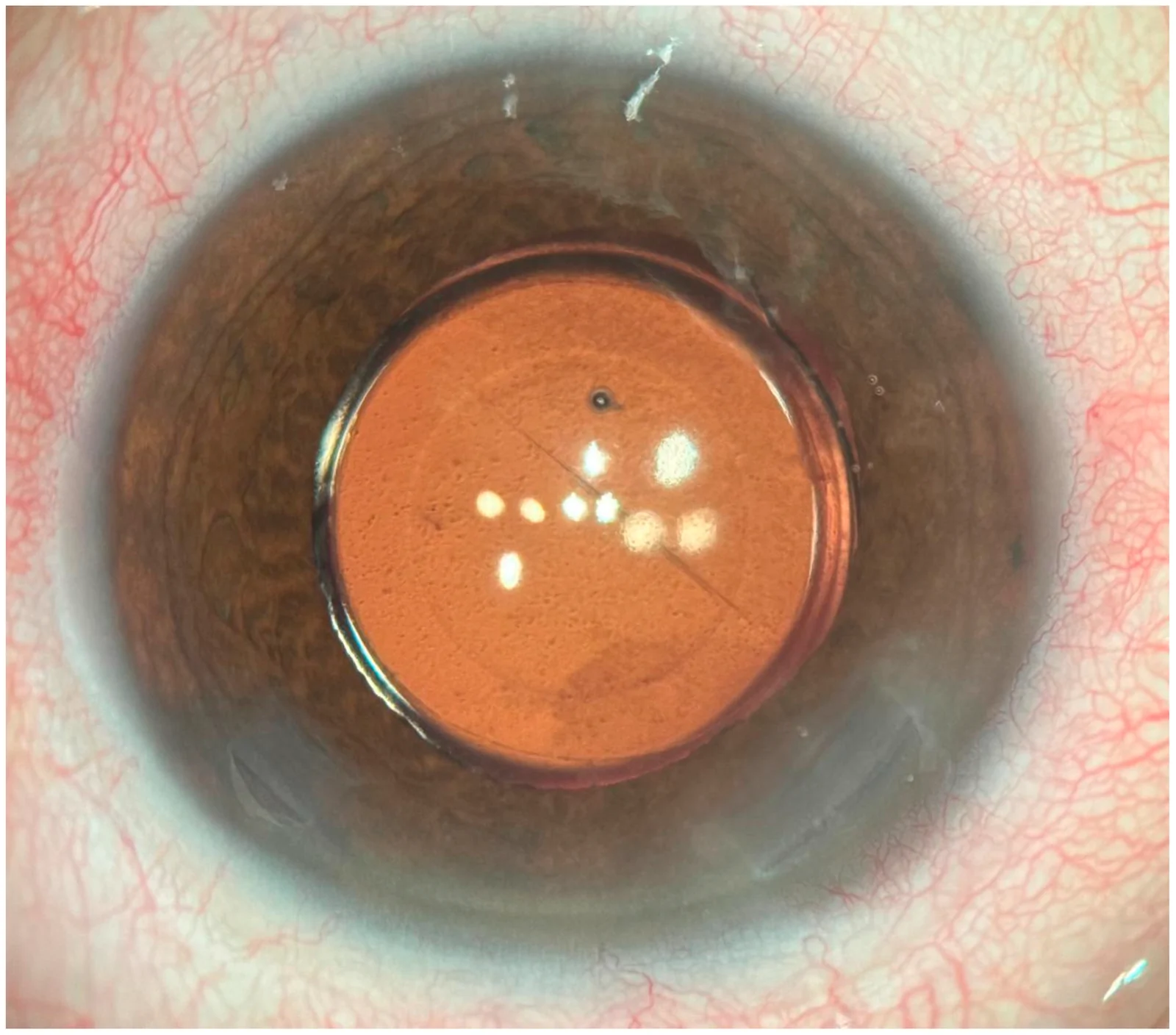
Slit-lamp appearance of a clinically centered Extend Bessel-optics intraocular lens at long-term follow-up (36–48 months) after implantation.

**Figure 2:**
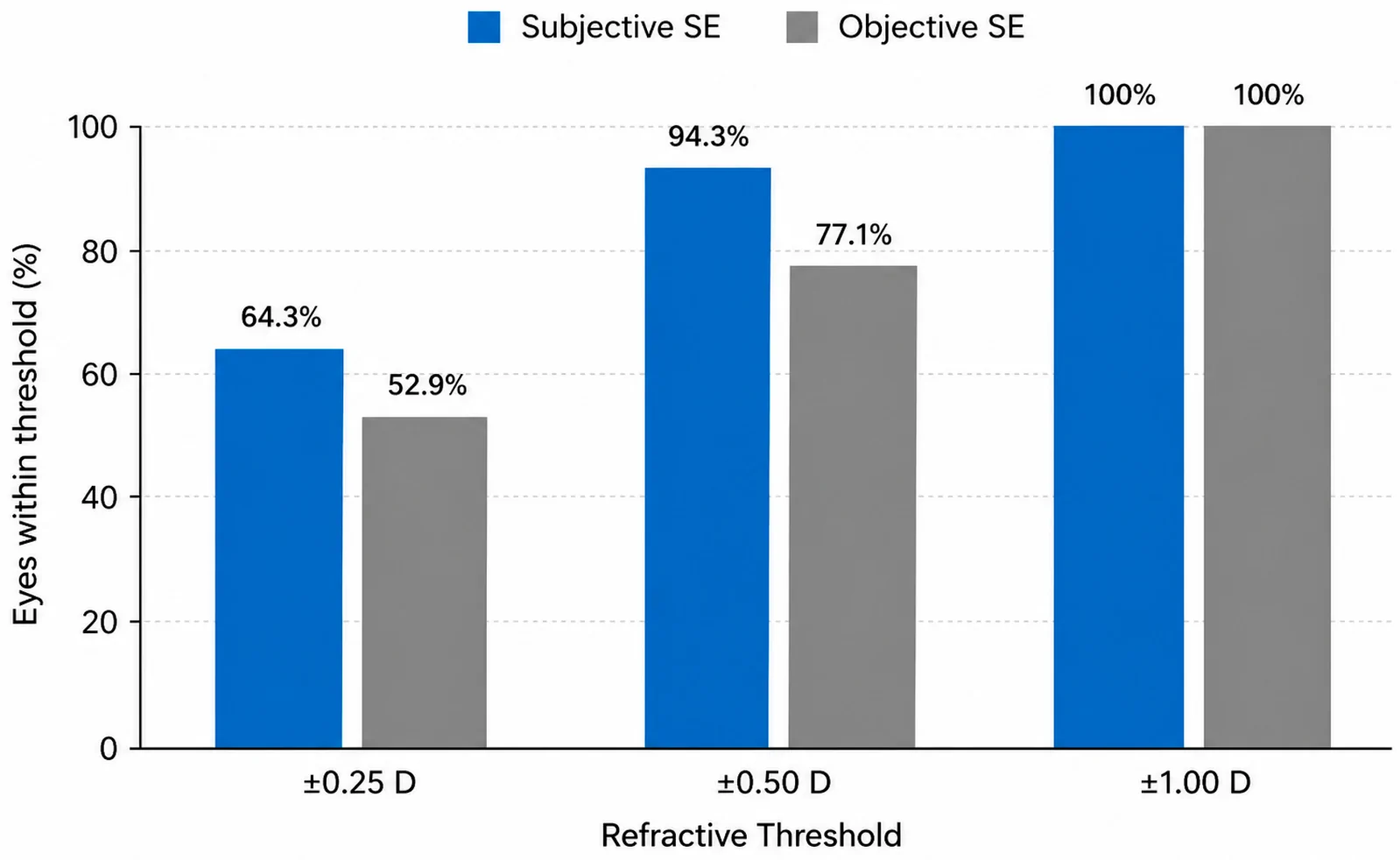
Postoperative refractive accuracy relative to emmetropia at long-term follow-up (36–48 months). Bars represent the proportion of eyes within ±0.25, ±0.50, and ±1.00 D of emmetropia according to subjective and objective postoperative spherical equivalent. (D, diopters; SE, spherical equivalent).

**Figure 3:**
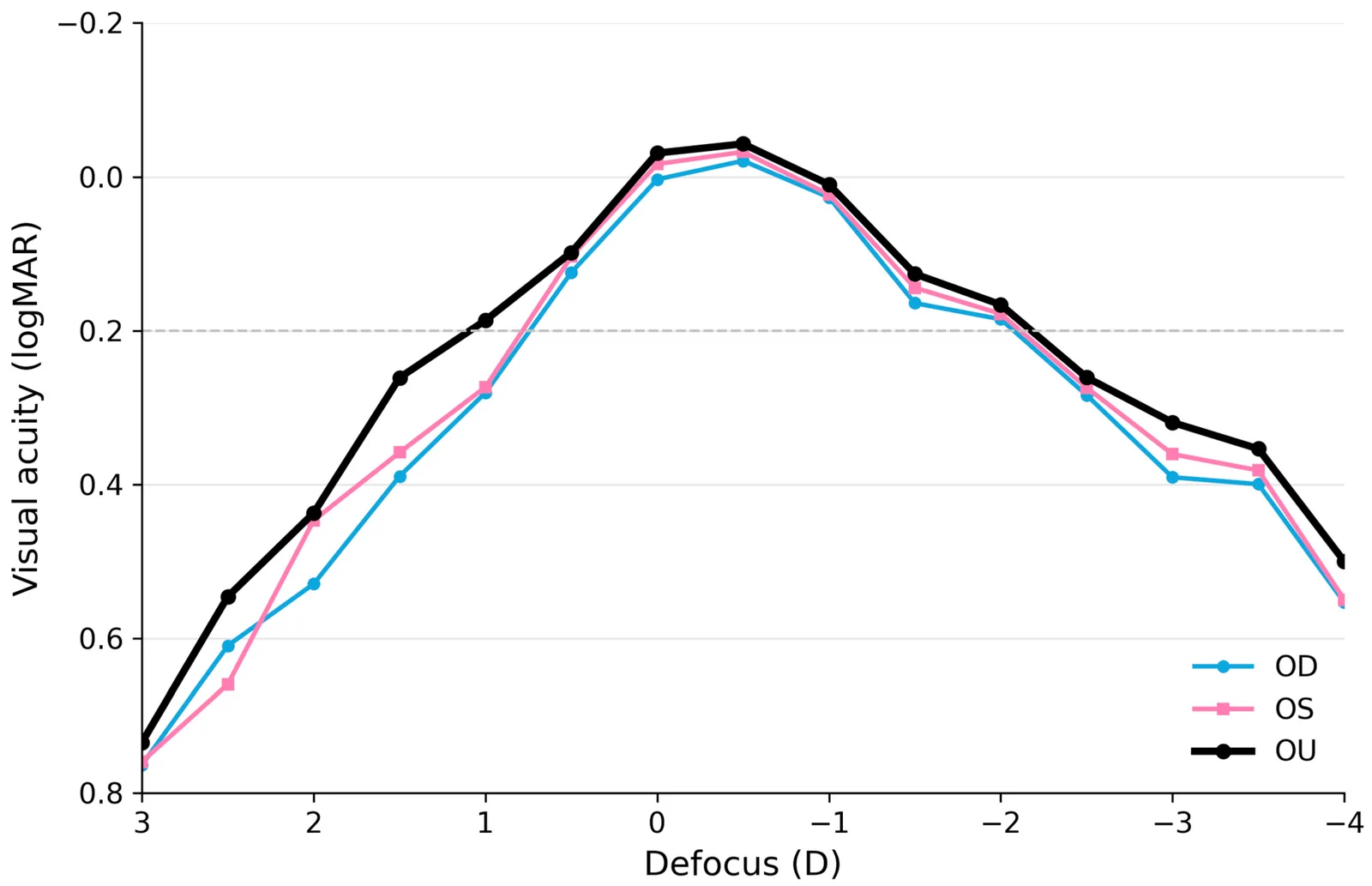
Mean monocular (OD and OS) and binocular (OU) defocus curves at long-term follow-up (36–48 months) after bilateral implantation of the Extend Bessel-optics intraocular lens. The plotted mean values were verified against the final analytic workbook. (OD, right eye; OS, left eye; OU, binocular vision).

**Table 1:** Long-Term Clinical Outcomes (36–48 Months).

Variable	Mean ± SD *
Subjective spherical equivalent (D)	−0.17 ± 0.27
Objective spherical equivalent (D)	−0.31 ± 0.30
UDVA (logMAR)	0.04 ± 0.05
CDVA (logMAR)	−0.03 ± 0.04
UIVA 66 cm (logMAR)	0.14 ± 0.07
DCIVA 66 cm (logMAR)	0.10 ± 0.06
UNVA 40 cm (logMAR)	0.24 ± 0.10
DCNVA 40 cm (logMAR)	0.17 ± 0.08
Binocular UDVA (logMAR)	−0.03 ± 0.05
Binocular UIVA 66 cm (logMAR)	0.12 ± 0.09
Binocular UNVA 40 cm (logMAR)	0.26 ± 0.12
Photopic pupil (mm)	2.28 ± 0.13
Mesopic pupil (mm)	2.80 ± 0.22

* Values are presented as mean ± SD. CDVA, corrected distance visual acuity; DCIVA, distance-corrected intermediate visual acuity; DCNVA, distance-corrected near visual acuity; D, diopters; IOL, intraocular lens; logMAR, logarithm of the minimum angle of resolution; SE, spherical equivalent; UDVA, uncorrected distance visual acuity; UIVA, uncorrected intermediate visual acuity; UNVA, uncorrected near visual acuity.

**Table 2:** Patient-Level Longitudinal Refractive and Visual Outcomes Between One Year and Long-Term Follow-up.

Variable	1 Year	Long-Term (36–48 Months) *	Mean Paired Change (95% CI)	*p* Value
Subjective SE (D)	−0.204 ± 0.208	−0.175 ± 0.204	+0.029 ± 0.097 (−0.005 to +0.062)	0.114
Objective SE (D)	−0.239 ± 0.196	−0.291 ± 0.208	−0.052 ± 0.094 (−0.084 to −0.019)	0.003
UDVA (logMAR)	0.029 ± 0.039	0.038 ± 0.037	+0.009 ± 0.016 (+0.003 to +0.014)	0.002
UIVA at 66 cm (logMAR)	0.113 ± 0.050	0.140 ± 0.051	+0.027 ± 0.029 (+0.017 to +0.037)	<0.001
UNVA at 40 cm (logMAR)	0.210 ± 0.066	0.235 ± 0.060	+0.025 ± 0.029 (+0.015 to +0.035)	<0.001

* Values in this table are patient-level bilateral averages (n = 35) and are presented as mean ± SD. Mean changes are long-term minus one-year values. Inferential *p* values are from Wilcoxon signed-rank tests. The 95% confidence interval for mean paired change is reported for effect-size precision. Positive logMAR changes indicate worse visual acuity. Holm-adjusted *p* values for the four secondary longitudinal outcomes were 0.0046 (objective SE), 0.0046 (UDVA), 0.00016 (UIVA), and 0.00021 (UNVA). D, diopters; SE, spherical equivalent; UDVA, uncorrected distance visual acuity; UIVA, uncorrected intermediate visual acuity; UNVA, uncorrected near visual acuity.

**Table 3:** Visual Function, Spectacle Independence, and Patient Satisfaction at Long-Term Follow-up.

Value	Variable
**Visual Function**	
98.9 ± 2.5	Mean VF-14
100	Median
91.1–100	Range
28 (80.0)	Maximum score (score = 100)
**Spectacle independence**	
Never use spectacles	Vision
33 (94.3%)	Distance
34 (97.1%)	Intermediate
33 (94.3%)	Near
**Overall Satisfaction**	
n (%)	Variable
34 (97.1)	Very satisfied
1 (2.9)	Satisfied
35 (100)	Would undergo the same procedure again

**Table 4:** Visual Symptoms (Dysphotopsia) at Long-Term Follow-up, n = 35.

Symptom	None	Mild	Moderate	Severe
Halos	30 (85.7%)	4 (11.4%)	1 (2.9%)	0 (0.0%)
Glare	34 (97.1%)	1 (2.9%)	0 (0.0%)	0 (0.0%)
Blurred distance vision	32 (91.4%)	3 (8.6%)	0 (0.0%)	0 (0.0%)
Blurred near vision	32 (91.4%)	3 (8.6%)	0 (0.0%)	0 (0.0%)
Night vision difficulty	32 (91.4%)	3 (8.6%)	0 (0.0%)	0 (0.0%)
Monocular diplopia	35 (100.0%)	0 (0.0%)	0 (0.0%)	0 (0.0%)
Binocular diplopia	35 (100.0%)	0 (0.0%)	0 (0.0%)	0 (0.0%)
Depth perception difficulty	35 (100.0%)	0 (0.0%)	0 (0.0%)	0 (0.0%)
Color vision difficulty	35 (100.0%)	0 (0.0%)	0 (0.0%)	0 (0.0%)

## Data Availability

The anonymized dataset supporting the findings of this study is publicly available in the Zenodo repository at https://doi.org/10.5281/zenodo.21328601.

## Supplementary Materials

The following supporting information can be downloaded at: https://www.mdpi.com/article/10.3390/medicina62101883/s1. Table S1: Study-specific questionnaire domains and response categories. The questionnaire was administered in Spanish. This table summarizes the study domains and response categories represented in the analytic dataset; it is not intended as a verbatim reproduction of the original patient-facing form; Table S2: Exact aggregate mean visual-acuity values used for the final defocus curves. Values are mean logMAR acuity from the final analytic workbook. OD = right eye; OS = left eye; OU = binocular. The retained analytic workbook contains aggregate means but not the corresponding individual-level defocus observations; therefore, SD/error bars cannot be reconstructed from this workbook alone.

## Abbreviations

The following abbreviations are used in this manuscript:

CDVA	corrected distance visual acuity
D	diopters
DCIVA	distance-corrected intermediate visual acuity
DCNVA	distance-corrected near visual acuity
IOL	Intraocular lens
logMAR	logarithm of the minimum angle of resolution
SE	spherical equivalent
UDVA	uncorrected distance visual acuity
UIVA	uncorrected intermediate visual acuity
UNVA	uncorrected near visual acuity
