# Supplementary material for: Three-Year Functional Vision, Longitudinal Refractive Outcomes, and Patient-Reported Outcomes After Bilateral Implantation of the Extend Bessel-Optics Intraocular Lens: An Independent Real-World Ambispective Study
Source: Medicina (Kaunas). 2026 Sep 29;62(10):1883. doi: 10.3390/medicina62101883 (PMC13706877; doi:10.3390/medicina62101883)
Supplement: Supplementary file 1 [file medicina-62-01883-s001.zip › medicina-4466181-supplementary.pdf]

Supplementary Material

Extend Bessel-Optics IOL: 3- to 4-Year Ambispective Study

Table S1. Study-specific questionnaire domains and response categories.

The questionnaire was administered in Spanish. This table summarizes the study domains and response categories represented in the analytic dataset; it is not intended as a verbatim reproduction of the original patient-facing form.

Domain	Item	Response categories
Visual phenomena	Halos	None / Mild / Moderate / Severe
Visual phenomena	Glare	None / Mild / Moderate / Severe
Visual phenomena	Blurred distance vision	None / Mild / Moderate / Severe
Visual phenomena	Blurred near vision	None / Mild / Moderate / Severe
Visual phenomena	Monocular diplopia	None / Mild / Moderate / Severe
Visual phenomena	Binocular diplopia	None / Mild / Moderate / Severe
Visual phenomena	Night-vision difficulty	None / Mild / Moderate / Severe
Visual phenomena	Depth-perception difficulty	None / Mild / Moderate / Severe
Visual phenomena	Color-vision difficulty	None / Mild / Moderate / Severe
Spectacle use	Distance	Never / Sometimes / Nearly always
Spectacle use	Intermediate	Never / Sometimes / Nearly always
Spectacle use	Near	Never / Sometimes / Nearly always
Overall satisfaction	Overall surgical outcome	Very satisfied / Satisfied / Less satisfied / Unsatisfied
Willingness to repeat	Would undergo the same procedure again	Yes / No

Table S2. Exact aggregate mean visual-acuity values used for the final defocus curves.

Values are mean logMAR acuity from the final analytic workbook. OD = right eye; OS = left eye; OU = binocular. The retained analytic workbook contains aggregate means but not the corresponding individual-level defocus observations; therefore, SD/error bars cannot be reconstructed from this workbook alone.

Defocus (D)	OD mean logMAR	OS mean logMAR	OU mean logMAR
+3.00	0.763	0.759	0.734
+2.50	0.609	0.659	0.546
+2.00	0.529	0.447	0.437
+1.50	0.389	0.358	0.261
+1.00	0.281	0.273	0.187
+0.50	0.124	0.104	0.098
+0.00	0.003	-0.017	-0.031
-0.50	-0.021	-0.032	-0.043
-1.00	0.027	0.023	0.010
-1.50	0.164	0.144	0.126
-2.00	0.185	0.178	0.166
-2.50	0.284	0.274	0.261
-3.00	0.391	0.361	0.319
-3.50	0.400	0.382	0.354
-4.00	0.553	0.550	0.500
